# Comparative effectiveness of chest ultrasound, chest X-ray and computer-aided diagnostic (CAD) for tuberculosis diagnosis in low-resource setting: study protocol for a cross-sectional study from Ethiopia

**DOI:** 10.3389/fpubh.2024.1476866

**Published:** 2024-11-28

**Authors:** Giacomo Guido, Worku Nigussa, Sergio Cotugno, Birhanu Kenate Sori, Flavio Antonio Bobbio, Berhanu Gulo, Luigi Pisani, Fabio Manenti, Mulugeta Miressa, Francesco Cavallin, Surra Abata, Francesco Vladimiro Segala, Abdi Reta, Ottavia Tulome, Giovanni Putoto, Roberta Iatta, Antonino Tuttolomondo, Nicola Veronese, Mario Barbagallo, Annalisa Saracino, Francesco Di Gennaro

**Affiliations:** ^1^Clinic of Infectious Diseases, Department of Precision and Regenerative Medicine and Ionian Area – (DiMePRe-J), University of Bari "Aldo Moro", Bari, Italy; ^2^Doctors with Africa CUAMM, Wolisso, Ethiopia; ^3^Oromia Regional Health Bureau, Addis Ababa, Ethiopia; ^4^Department of Precision-Regenerative Medicine and Jonic Area (DiMePRe-J), Section of Anesthesiology and Intensive Care Medicine, University of Bari "Aldo Moro", Bari, Italy; ^5^Doctors with Africa CUAMM, Padua, Italy; ^6^Independent Statistician, Solagna, Italy; ^7^Interdisciplinary Department of Medicine, University of Bari, Bari, Italy; ^8^Geriatric Unit, Department of Medicine, University of Palermo, Palermo, Italy

**Keywords:** CAD, tuberculosis, chest ultrasound, Ethiopia, Africa, diagnosis, CAD4TB, pulmonary tuberculosis

## Abstract

**Introduction:**

Early and accurate diagnosis of pulmonary tuberculosis (TB) is crucial for timely treatment and prevention of transmission, but diagnostic challenges persist due to complex symptoms and limitations in diagnostic tools. Chest X-ray (CXR) is the standard imaging modality, but its sensitivity and specificity may vary. Recently, some promising alternatives emerged such as chest ultrasonography (CUS) – particularly valuable in resource-limited settings – and computer-aided diagnosis (CAD) systems – helping clinicians in the reading and interpretation of the CXR. However, direct comparisons of CUS, CXR, and CAD score in TB diagnosis are limited.

**Methods and analysis:**

This cross-sectional study will assess the diagnostic effectiveness of CUS in diagnosing TB compared to CXR and CAD score among index cases and household contacts. The study will be conducted at Wolisso St. Luke Hospital (Wolisso, Ethiopia). Index cases will be subjects with diagnosis of pulmonary tuberculosis within 7 days. Household contacts will be identified by administering a screening questionnaire to index cases. They will undergo CXR as for standard of care and consequent CAD analysis and CUS. The anticipated sample size is 136 subjects. The common accuracy metrics (sensitivity, specificity, positive and negative predictive values) will be calculated.

**Ethics and dissemination:**

The protocol was approved by the Oromia Health Bureau Research Ethics Committee (BFO/MBTFH/1-16/1908). All information obtained will be confidential. Selected investigators will have access to data, while international partners will sign a dedicated Data Protection Agreement. Eligible subjects will receive a brief information about the study before being asked to participate and they will provide a written informed consent. Results will be conveyed to stakeholders and disseminated through conferences and peer-reviewed journals.

**Clinical trial registration:**

NCT06409780, https://clinicaltrials.gov.

## Highlights

- This study will assess the effectiveness of CUS in diagnosing TB in a broad population, as opposed to targeted populations such as people living with HIV.
- This study will also involve household contacts in a deeper screening program.
- CUS is easy to perform without risks for the patient.
- Index cases will be diagnosed using molecular tests instead of cultural tests.
- CUS performance may vary according to operator’s skills.
- Cavitations are less frequently observed with CUS.

## Introduction

Pulmonary Tuberculosis (TB) is caused by the *M. tuberculosis complex*, a slow-growing bacteria with a particular cell wall composition that confers resistance to the main antibiotics. Despite being one of the oldest known diseases, TB remains one of the biggest concerns in global health, especially in at-risk populations such as HIV/AIDS subjects. In 2022, over 1.2 million deaths were attributed to TB globally, with 167,000 of these cases involving coinfection with HIV (1). The clinical presentation of TB can vary according to the site of infection and the individual’s immune status. TB typically affects the lungs and is associated with persistent cough, low-grade fever, night sweats, weight loss and hemoptysis, while extrapulmonary TB may affect any other site of the body, with different manifestations and treatment strategies (2). The main determinant for TB is poverty, and most people developing TB live in low-middle income countries (LMIC) where healthcare resources are often limited (3, 4). Further risk factors include diabetes, malnourishment, and alcohol use disorders (5, 6). Recently, the COVID-19 pandemic has constrained the TB eradication programs planned by the World Health Organization due to diagnostic delay (7), along with climate change and internal and international conflicts.

Early and accurate TB diagnosis is crucial for timely initiation of treatment and prevention of transmission, yet it presents challenges due to several factors including complexity of symptoms and limitations in diagnostic tools (8). The widespread adoption of molecular tests such as GeneXpert has significantly simplified and enhanced the sensitivity of microbiological diagnosis for TB, to the extent that it has replaced smear microscopy as the diagnostic gold standard in many national guidelines, especially in LMICs (9). Furthermore, GeneXpert provides clinicians with a rapid assessment of TB strain susceptibility, enabling timely initiation of appropriate treatment. For instrumental diagnosis of TB, chest X-ray (CXR) is the standard imaging modality for TB diagnosis, but its sensitivity and specificity can vary according to some factors such as disease stage and image quality (10). Computed tomography scans may offer higher resolution and details, but availability and accessibility can be limited particularly in resource-constrained settings (11, 12). In Ethiopia, a recent study on the facilitators of pulmonary TB diagnosis emphasized the importance of integrating radiographic screening with symptom-based screening in health facilities, while acknowledging the high cost of such implementation (13). The constraints in implementing CXR-based screening included the requirement for radiological equipment (which may not always be accessible), the exposure to ionizing radiation (with potential risks for vulnerable populations such as children and pregnant women), and the shortage of personnel trained in acquiring and interpreting quality images in the health facilities.

In an era marked by the proliferation of artificial intelligence, efforts have been made to overcome the shortage of trained personnel (14). Recently, a software for radiographic image analysis (CAD) has been introduced to provide clinicians with a probability score for TB detection (15). Furthermore, chest ultrasonography (CUS) has emerged as a promising adjunctive tool in TB diagnosis, especially in at-risk populations (16). Its advantages include portability, lack of radiation exposure, and potential for bedside use, making it particularly valuable in LMICs where access to advanced imaging techniques may be limited (15, 17). The initial evidence has suggested that CUS may offer high sensitivity in detecting microbiologically confirmed TB among adults, but available data are still limited (18, 19). Furthermore, limited research has directly compared the diagnostic performance of CUS, CXR, and CAD score in the diagnosis of TB, and no evidence has been produced for household contacts of index cases (20).

Understanding the comparative effectiveness of these diagnostic modalities is essential for optimizing TB diagnosis strategies and improving patient outcome, especially in high-risk populations such as household contacts who are at increased risk of TB transmission (21, 22). Therefore, this study aims to fill this gap by evaluating the diagnostic accuracy of CUS, CXR, and CAD score in identifying TB in index cases and household contacts. The findings may provide valuable insights into the effectiveness of these modalities and inform the development of contextually appropriate TB diagnostic strategies to improve patient outcome.

## Methods and analysis

### Study design

This study will employ a cross-sectional design to compare the diagnostic effectiveness of CUS with CXR and CAD score in identifying TB among index cases and household contacts.

### Study setting

The study will be conducted at St. Luke Hospital in Wolisso (Ethiopia), which is a second level, not-for-profit hospital covering a catchment area of about 1.2 million inhabitants. The Medical Ward has 23 beds and accounts for around 1,800 admissions every year. Wolisso is the largest city in the South-western Shewa area of the Oromya Region. According to WHO TB Report 2023, in Ethiopia the incidence of TB is 126 cases every 100,000/people, of which 4.9 cases of co-infection HIV/TB every 100,000 people, 1.6 case of rifampicin-resistant/multidrug resistant TB every 100 k people (1,1% of all new cases), with TB case fatality ratio of 15%.

### Participant and eligibility criteria

Eligible subjects will be all consecutive index cases during the study period and their household contacts. Index cases will be identified following a microbiological diagnosis of pulmonary tuberculosis using GeneXpert (Xpert MTB/RIF assay) within 7 days from the beginning of anti-tubercular treatment, in accordance with the standard of care in the Outpatient Department or the Medical Ward at St. Luke hospital. Household contacts will be identified by administering a screening questionnaire to index cases. Household contacts are defined as individuals who live in the same household as the index case or who had close and prolonged interactions with the index case during the infectious period. To minimize recruitment attrition, research assistants will reach out to household contacts and offer transportation to St. Luke hospital to facilitate their participation in the screening program. Eligible subjects of both sexes and aged over 5 years will be included, and all participants will be asked to provide informed consent. Exposure to any antitubercular treatment prior 7 days than the enrollment and refusing consent to participate will be the only exclusion criteria.

### Procedures

After enrollment, all participants will undergo imaging examination including CUS and CXR according to standard protocols. Considering the high interoperator variability of the ultrasounds, two expert and trained sonographers will perform CUS. In case of discordance, the final decision will rest with the senior sonographer.

During CUS, the participant will stay in a supine or seated position, exposing the chest area for ultrasound examination. A thin layer of ultrasound gel will be applied to the skin to facilitate acoustic coupling and improve image quality. During CXR, the participant will stay in an upright or standing position, facing the X-ray machine. Images will be captured in the posteroanterior view, ensuring adequate visualization of the chest area. The acquired chest X-ray images will be transferred to the CAD4TB software platform (15), which may highlight regions of interest and assess a score according to the findings.

Reference standard for TB diagnosis in household contacts will be based on microbiological confirmation according to local protocol (molecular diagnosis with GeneXpert MTB/RIF assay) or clinical-radiological criteria.

Biological specimens will be collected and managed according to TB local standard of care. A volume of 5 to 10 mL of sputum is considered adequate for the purpose and there is no advantage in collecting a larger volume. The sample should contain recently discharged material from the bronchial tree with minimal saliva content. Samples will be handled as per local routine laboratory procedures and will not be stored for research purposes.

All household contacts will also be screened for the presence of any symptom suggestive of TB, including cough of any duration, hemoptysis, fever, poor weight gain or weight loss, night sweats, chest pain, and shortness of breath.

Standard of care for index cases and household contacts will be provided according to the Ethiopian National Guidelines for Tuberculosis. The study procedures are not expected to interfere with the routinary activities related with the admission and the care of the patients.

### Outcome measures

The primary outcome measures will include the sensitivity and the specificity of CUS, CXR, and CAD in TB diagnosing. The secondary outcome measures will include (i) the positive predictive value and the negative predictive value of CUS, CXR, and CAD in diagnosing TB, and (ii) the concordance between CUS, CXR, and CAD in identifying TB cases.

### Data collection

Data collection will include demographic and clinical information (such as age, sex, symptoms and TB risk factors), and data from CUS, CXR and CAD. Data collection will be performed by previously trained data collectors. All data will be anonymized and stored into a dedicated, password-protected database.

### Sample size

The sample size calculation is based on the anticipated sensitivity (0.80) and specificity (0.80) of the diagnostic modalities (CUS, CXD, CAD). A paired design will be used to test whether the sensibility (and specificity) of the experimental modality (CUS) is non-inferior to sensibility (and specificity) of the reference modalities (CXD and CAD separately), with a non-inferiority difference bound of −0.10. With a type I error of 5% and a power of 90%, 110 subjects are required to exclude a difference in favor of the reference group of more than 0.10. The final sample size is increased to 136 subjects to take into account the adjustment for multiple testing, both index cases and household contacts. The calculation was performed according to Liu et al. (23) and was carried out using R 4.3 (R Foundation for Statistical Computing, Vienna, Austria) (24).

### Statistical analysis

Two interim analyses are planned at 3 and 6 months of enrollment to check the assumptions on sensitivity and specificity of CUS, CXR and CAD in diagnosing TB. The interim analyses will not include any formal statistical testing. Adjustments to sample size and duration of enrolling period may be made according to the indications from the interim assessments. There are no stopping guidelines for harm and/or futility as none are expected. Categorical data will be summarized as absolute and relative frequencies. Numerical data will be summarized using mean and standard deviation (SD), or median and interquartile range (IQR). In accuracy investigation, the standard measures will be calculated (sensitivity, specificity, positive predictive value, negative predictive value). The non-inferiority hypothesis will be tested using a RMLE-based score test according to Liu et al. (23) Adjustment for multiple testing will be performed according to Benjamini-Hochberg procedure. Concordance between CUS and CXR, and between CUS and CAD will be assessed using Cohen’s kappa and Gwet’s AC1. Comparisons between variables will be performed with exploratory purpose using Pearson’s or Spearman’s correlation coefficients, Student’s t-test, paired Student’s t-test, Mann–Whitney test, Wilcoxon test, Chi Square test, or Fisher’s test, as appropriate. Estimates will be reported with 95% confidence intervals where appropriate. Statistical significance will be set at 5%. The statistical analysis will be carried out using R 4.3 (R Foundation for Statistical Computing, Vienna, Austria) (24).

### Patient and public involvement

Patients and public were not involved in the design and design, or conduct, or reporting of our research. Patients and public will be involved in the dissemination plans of our research.

## Ethics and dissemination

The protocol was approved by the Oromia Health Bureau Research Ethics Committee (BFO/MBTFH/1–16/1908). This study will be conducted in accordance with the ethical principles outlined in the Declaration of Helsinki.

Eligible subjects will be informed about the study before enrollment and participants will provide a written informed consent in an appropriate language (English/Oromifa). In case of illiteracy, a literature witness will sign, and the illiterate participant will provide his/her fingerprint. Participation in the study is voluntary and participants may withdraw at any time. All subjects will be informed that refusal to participate or withdrawal from the study will not affect their care.

All information obtained will be confidential, and all data will be anonymized and stored into a dedicated, password-protected database. Selected investigators will have access to the data, and international partners will sign a dedicated Data Protection Agreement.

The risks associated with participating in this study include minimal radiation exposure from CXR and potential discomfort during imaging procedures. However, the potential benefits of this study include contributing to the advancement of TB diagnostic strategies and early detection of TB cases, and indirectly contributing to improved patient outcome. There are no direct benefits to participants in this study.

The findings will be conveyed to the relevant stakeholders such as governmental health institutions, hospital management, healthcare staff, local community and decision-makers. The findings will be disseminated in scientific and community conferences, and will be reported in a peer-reviewed journal.

## References

[1] WHO. (2023) TB report 2023, WHO. Available online at: https://iris.who.int/bitstream/handle/10665/373828/9789240083851-eng.pdf?sequence=1

[2] Cotugno S Guido G Manco Cesari G Ictho J Lochoro P Amone J . Cardiac tuberculosis: a case series from Ethiopia, Italy, and Uganda and a literature review. Am J Trop Med Hyg. (2024) 110:795–804. doi: 10.4269/ajtmh.23-0505, PMID: 38412542 PMC10993843

[3] Di Gennaro F Cotugno S Fasano M Ricciardi A Ronga L Lattanzio R . High risk of unsuccessful treatment outcome in migrant population with tuberculosis: data from three Italian hospitals. Front Public Health. (2023) 10:1024474. doi: 10.3389/fpubh.2022.1024474, PMID: 36703820 PMC9871451

[4] Arega B Tilahun K Minda A Agunie A Mengistu G. Prevalence rate of undiagnosed tuberculosis in the community in Ethiopia from 2001 to 2014: systematic review and meta-analysis. Arch Public Health. (2019) 77:33. doi: 10.1186/s13690-019-0360-2, PMID: 31333842 PMC6622003

[5] Pizzol D Di Gennaro F Chhaganlal KD Fabrizio C Monno L Putoto G . Prevalence of diabetes mellitus in newly diagnosed pulmonary tuberculosis in Beira. Mozambique Afr Health Sci. (2017) 17:773–9. doi: 10.4314/ahs.v17i3.20, PMID: 29085405 PMC5656213

[6] Duarte R Lönnroth K Carvalho C Lima F Carvalho ACC Muñoz-Torrico M . Tuberculosis, social determinants and co-morbidities (including HIV). Pulmonology. (2018) 24:115–9. doi: 10.1016/j.rppnen.2017.11.003, PMID: 29275968

[7] Di Gennaro F Gualano G Timelli L Vittozzi P Di Bari V Libertone R . Increase in tuberculosis diagnostic delay during first wave of the COVID-19 pandemic: data from an Italian infectious disease referral hospital. Antibiotics (Basel). (2021) 10:272. doi: 10.3390/antibiotics10030272, PMID: 33800406 PMC7998965

[8] Danchuk SN Solomon OE Kohl TA Dreyer V Barilar I Utpatel C . Challenging the gold standard: the limitations of molecular assays for detection of Mycobacterium tuberculosisheteroresistance. Thorax. (2024) 79:670–5. doi: 10.1136/thorax-2023-220202, PMID: 38286614 PMC11187393

[9] Ministry of Health Ethiopia. Guidelines for clinical and programmatic management of TB, TB/HIV, DR-TB and leprosy in Ethiopia. 7th ed. Addis Ababa: Ministry of Health Ethiopia (2021).

[10] Acharya V Dhiman G Prakasha K Bahadur P Choraria A M S . AI-assisted tuberculosis detection and classification from chest X-rays using a deep learning normalization-free network model. Comput Intell Neurosci. (2022) 2022:2399428–19. doi: 10.1155/2022/2399428, PMID: 36225551 PMC9550434

[11] Skoura E Zumla A Bomanji J. Imaging in tuberculosis. Int J Infect Dis. (2015) 32:87–93. doi: 10.1016/j.ijid.2014.12.007 25809762

[12] Weldemhret L. Burden of pulmonary tuberculosis and challenges related to tuberculosis detection in Ethiopia. J Infect Dev Ctries. (2023) 17:578–82. doi: 10.3855/jidc.13169, PMID: 37279415

[13] Abraham Y Manyazewal T Amdemariam Z Petros H Ayenadis F Mekonen H . Facilitators and barriers to implementing chest radiography in tuberculosis systematic screening of clinically high-risk groups in Ethiopia: a qualitative study. SAGE open medicine. (2024) 12:20503121241233232. doi: 10.1177/20503121241233232, PMID: 38379811 PMC10878208

[14] Segala FV Nigussa W Guido G Kenate B Facci E Tsegaye A . Active close contact investigation of tuberculosis through computer-aided detection and stool Xpert MTB/RIF among people living in Oromia region, Ethiopia (CADOOL study): protocol for a prospective, cross-sectional study. BMJ Open. (2023) 13:e074968. doi: 10.1136/bmjopen-2023-074968, PMID: 38135314 PMC10749013

[15] Abuzerr S Zinszer K. Computer-aided diagnostic accuracy of pulmonary tuberculosis on chest radiography among lower respiratory tract symptoms patients. Front Public Health. (2023) 11:1254658. doi: 10.3389/fpubh.2023.1254658, PMID: 37965525 PMC10641698

[16] Bobbio F Di Gennaro F Marotta C Kok J Akec G Norbis L . Focused ultrasound to diagnose HIV-associated tuberculosis (FASH) in the extremely resource-limited setting of South Sudan: a cross-sectional study. BMJ Open. (2019) 9:e027179. doi: 10.1136/bmjopen-2018-027179, PMID: 30944140 PMC6500283

[17] Suttels V Du Toit JD Fiogbé AA Wachinou AP Guendehou B Alovokpinhou F . Point-of-care ultrasound for tuberculosis management in sub-Saharan Africa-a balanced SWOT analysis. Int J Infect Dis. (2022) 123:46–51. doi: 10.1016/j.ijid.2022.07.009, PMID: 35811083

[18] Fentress M Ugarte-Gil C Cervantes M Rivas D Moore D Caliguiri P . Lung ultrasound findings compared with chest X-ray findings in known pulmonary tuberculosis patients: a cross-sectional study in Lima. Peru Am J Trop Med Hyg. (2020) 103:1827–33. doi: 10.4269/ajtmh.20-0542, PMID: 32815504 PMC7646758

[19] Di Gennaro F Pisani L Veronese N Pizzol D Lippolis V Saracino A . Potential diagnostic properties of chest ultrasound in thoracic tuberculosis-a systematic review. Int J Environ Res Public Health. (2018) 15:2235. doi: 10.3390/ijerph15102235, PMID: 30322009 PMC6210728

[20] Fentress M Henwood PC Maharaj P Mitha M Khan D Caligiuri P . High sensitivity of ultrasound for the diagnosis of tuberculosis in adults in South Africa: a proof-of-concept study. PLOS Glob Public Health. (2022) 2:e0000800. doi: 10.1371/journal.pgph.0000800, PMID: 36962607 PMC10021214

[21] Cozzi D Bartolucci M Giannelli F Cavigli E Campolmi I Rinaldi F . Parenchymal Cavitations in pulmonary tuberculosis: comparison between lung ultrasound, chest X-ray and computed tomography. Diagnostics (Basel). (2024) 14:522. doi: 10.3390/diagnostics14050522, PMID: 38472994 PMC10930917

[22] Rea G Sperandeo M Lieto R Bocchino M Quarato CMI Feragalli B . Chest imaging in the diagnosis and Management of Pulmonary Tuberculosis: the complementary role of thoracic ultrasound. Front Med (Lausanne). (2021) 8:753821. doi: 10.3389/fmed.2021.753821, PMID: 34957142 PMC8703038

[23] Liu JP Hsueh HM Hsieh E Chen JJ. Tests for equivalence or non-inferiority for paired binary data. Stat Med. (2002) 21:231–45. doi: 10.1002/sim.1012 11782062

[24] R Core Team (2024). R: A language and environment for statistical computing. R Foundation for Statistical Computing, Vienna, Austria. Available online at: https://www.R-project.org/

