# Supplementary material for: Comparative effectiveness of chest ultrasound, chest X-ray and computer-aided diagnostic (CAD) for tuberculosis diagnosis in low-resource setting: study protocol for a cross-sectional study from Ethiopia
Source: Front Public Health. 2024 Nov 28;12:1476866. doi: 10.3389/fpubh.2024.1476866 (PMC11650444; doi:10.3389/fpubh.2024.1476866)
Supplement: Supplementary file 1 [file Data_Sheet_1.pdf]

Supplementary file

Chest ultrasound different patterns in Tuberculosis

PROCEDURES TO PERFORM AND ANALYSE CUS

The following instructions will guide the investigator in acquiring and saving lung ultrasound images. For the purposes, the terms "patient" and "participant" will be used interchangeably to indicate the subject who is receiving the ultrasound exam; the terms "operator," "investigator," and "examiner" will be used to indicate the person performing the ultrasound exam.

<u>Steps:</u>

1. Preparing the patient: confirm patient identity and informed consent in REDCap. Inform the patient, patient's nurse, and/or family. Reduce ambient lighting where possible.
2. Start the Butterfly lung ultrasound machine and connect to the iPad.
3. Open the Butterfly IQ App on the tablet. Follow the prompts on the screen to begin a new study (or click on the ultrasound icon at the bottom of the screen).
4. Choose the "lung" setting on the ultrasound machine. Click "Preset" → "Lung" → "Select". Select "Lung Protocol" in the actions menu (**Figure 1**).
5. *Examiner position*: typically, the examiner ought to stand on the patient's right side during the examination, using their right hand to hold the probe and their left hand to operate the console. The hand holding the probe should be placed on the patient's chest to ensure a steady view free from movement caused by the operator. If feasible, adjust the height of the bed to a comfortable level.
6. *Patient position*: If feasible, position the patient semi-upright. Alternatively, the examination can be performed with the patient

lying down or sitting. Ensure the thorax is exposed (or instruct the patient to do so).

7. Initiate the CUS examination by positioning the probe longitudinally over the area of interest, ensuring that the pointer is directed towards the patient's head. Locate the pleural line amidst the rib shadows. Keep in mind that the probe will be angled more obliquely along the sides of the lung.
8. Establish the depth at 10 cm initially, and then fine-tune it until the pleural line occupies one-third of the screen. To achieve this, simply swipe your finger up and down on the tablet, and a depth gauge will be displayed on the left side of the screen.
9. Ensure that you maintain an optimal technical view while documenting the most abnormal lung findings that are visible. For instance, if there are variations in patterns observed in the region of interest (e.g., 1L) across different intercostal spaces, please document the most severe pattern identified. The severity of patterns, in descending order, is as follows: C (most severe) > B2 > B1 > A (normal aeration).

Different patterns are described below.

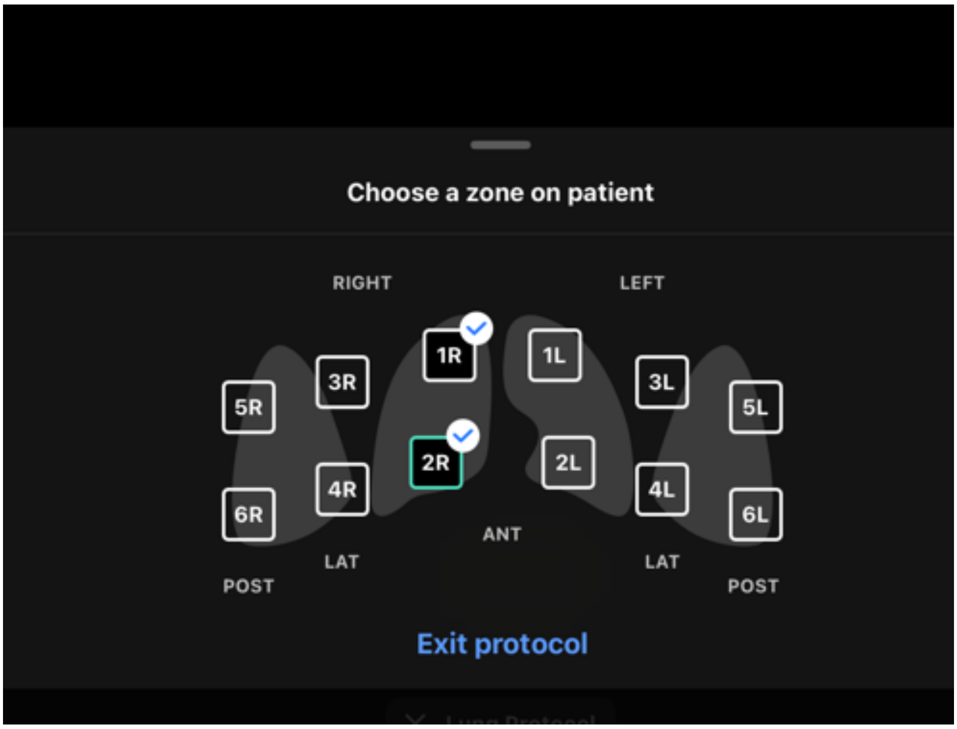

Figure 1. Lung Protocol interface on Butterfly IQ+

“**A-pattern**”: repeating horizontal A-lines parallel to the pleural line, suggesting normal aeration.

“**B1-pattern**”: Three or more vertical B-lines originating from the pleural line and extending to the bottom of the screen indicate a partial loss of aeration. Additionally, the B-lines are adequately spaced and occupy less than or equal to 50% of the pleural line.

“**B2-pattern**”: if B-lines cover ≥ 50% of the pleural line.

“**C-pattern**”: when the consolidation <u>is > 2cm</u> (i.e., consolidation, suggesting near-complete to complete loss of aeration).

“P-pattern”: if any subpleural consolidation/abnormal pleural line.

“E-pattern”: when presence of pleural effusion.

“U-pattern”: if unable to assess.
