# Supplementary material for: Comparative effectiveness of chest ultrasound, chest X-ray and computer-aided diagnostic (CAD) for tuberculosis diagnosis in low-resource setting: study protocol for a cross-sectional study from Ethiopia
Source: Front Public Health. 2024 Nov 28;12:1476866. doi: 10.3389/fpubh.2024.1476866 (PMC11650444; doi:10.3389/fpubh.2024.1476866)
Supplement: Supplementary file 2 [file Data_Sheet_2.pdf]

Demographics

Record ID	______________________
Index case or Household contact?	◯ Index<br>◯ Household contact
Index case name	______________________
Index OPD	______________________
OPD n°	______________________
First Name	______________________
Last Name	______________________
Date of Birth	______________________
Sex	◯ Male<br>◯ Female
Recruitment Date	______________________
Age at enrollment	______________________
Index case recruitment	◯ OPD<br>◯ Medical<br>◯ From Laboratory<br>◯ Outreach screening
How was index case diagnosed?	◯ Sputum GeneXpert<br>◯ Stool GeneXpert<br>◯ LAM<br>◯ Microscopy<br>◯ Other
Telephone number	______________________

district of residence	◯ Arsi Zone<br>◯ Bale Zone<br>◯ Borena Zone<br>◯ Buno Bedele Zone<br>◯ East Hararghe Zone<br>◯ East Shewa Zone<br>◯ East Welega Zone<br>◯ Guji Zone<br>◯ Horo Guduru Welega Zone<br>◯ Illubabor Zone<br>◯ Jimma Zone<br>◯ Kelam Welega Zone<br>◯ North Shewa Zone<br>◯ Southwest Shewa Zone<br>◯ West Arsi Zone<br>◯ West Guji Zone<br>◯ West Hararghe Zone<br>◯ West Shewa Zone<br>◯ West Welega Zone<br>◯ Adama Special Zone<br>◯ Jimma Special Zone<br>◯ Oromia Special Zone Surrounding Finfinne
Setting	◯ Urban<br>◯ Rural
village of residence	______________________________
What is the highest level of education that you attained?	◯ NO EDUCATION<br>◯ LOWER PRIMARY<br>◯ UPPER PRIMARY<br>◯ LOWER SECONDARY<br>◯ UPPER SECONDARY<br>◯ TERTIARY
Occupation	◯ Farmer<br>◯ Housewife<br>◯ Daily labourer<br>◯ Merchant<br>◯ Student<br>◯ No work<br>◯ Other
Religion	◯ Orthodox Christian<br>◯ Protestant Christian<br>◯ Muslim<br>◯ Catholic Christian<br>◯ Waaqeffanna<br>◯ Other
If other religions, specify	______________________________
Marital status	◯ Married<br>◯ Never married<br>◯ Divorced<br>◯ Widow/widower

CXR & CAD

Record ID	__________________________________

CXR

Was CXR properly performed?	◯ Yes<br>◯ No
If CXR was not performed, why?	◯ Bad internet connection<br>◯ Computer issues<br>◯ CXR was not performed by the patient<br>◯ TBcloud website did not work<br>◯ Other
if other reasons, specify	__________________________________
When was the CXR performed?	__________________________________
CXR findings	☐ Consolidation/Infiltrates<br>☐ Pleural effusion<br>☐ Cavity<br>☐ Nodules<br>☐ Atelectasis<br>☐ Other
Which lobes are involved?	☐ Right lower lobe<br>☐ Right medium lobe<br>☐ Right upper lobe<br>☐ Left upper lobe<br>☐ Left lower lobe
TIMIKA score	__________________________________<br>(20 punti per ogni lobo coinvolto, 40 punti se si vede cavità)

CAD4TB

Was CAD4TB performed?	◯ Yes<br>◯ No
If not, why?	◯ CXR not performed<br>◯ Low quality of CXR<br>◯ Issues with Thirona
CAD score?	__________________________________

Microbiologic tests

Record ID ______________________________

Sputum Xpert

Was the patient able to produce sputum sample?
◯ Yes
◯ No

Was sputum Xpert properly performed?
◯ Yes
◯ No

If sputum Xpert was not performed, please explain why ______________________________

Sputum Xpert result
◯ Positive
◯ Negative
◯ Invalid

Replication Load
◯ High
◯ Medium
◯ Low
◯ Unknown

Rifampicin Resistance
◯ Detected
◯ Undetected
◯ Indeterminate

Stool Xpert

Was the patient able to produce stool sample?
◯ Yes
◯ No

Was stool Xpert properly performed?
◯ Yes
◯ No

If stool Xpert was not performed, please explain why ______________________________

Stool Xpert result
◯ Positive
◯ Negative
◯ Invalid

Replication Load
◯ High
◯ Medium
◯ Low
◯ Unknown

Rifampicin Resistance
◯ Detected
◯ Undetected
◯ Indeterminate

TB Symptoms & Risk factors

Record ID	__________________________
Past history of TB?	◯ Yes<br>◯ No
If positive history of TB, did the patient completed the full course of ATT?	◯ Yes<br>◯ No
Pregnant status?	◯ Yes<br>◯ No
Known HIV status	◯ Positive<br>◯ Negative<br>◯ Unknown
If known person living with HIV, is the patient taking antiretroviral therapy?	◯ Yes, consistently<br>◯ Yes, irregularly<br>◯ Off-therapy
If patient is taking ART, how was his/her last HIV-RNA	◯ Undetectable<br>◯ Higher than 20cp/ml<br>◯ Unknown
CD4 count, if available	__________________________
Results of the HIV-test	◯ Positive<br>◯ Negative<br>◯ Not valid
Comorbidities	☐ COPD<br>☐ smoker<br>☐ ex-smoker (more than 1 year)<br>☐ diabetes<br>☐ malnutrition<br>☐ renal disease<br>☐ alcohol abuse<br>☐ immunosuppressive conditions other than HIV<br>☐ malignancy<br>☐ silicosis<br>☐ cirrhosis<br>☐ none
Cigarettes/beedies smoked per day	◯ 1-10 cigarettes or beedis/day<br>◯ 11-20 cigarettes or beedis/day<br>◯ > 30 cigarettes or beedis/day
Is the patient SYMPTOMATIC for TB?	◯ Yes<br>◯ No

Which symptoms does the patient refer?	☐ cough<br>☐ night sweats<br>☐ weight loss<br>☐ fever<br>☐ hemoptysis<br>☐ dyspnea<br>☐ chest pain<br>☐ others<br>☐ none
If other symptoms, specify	__________________________
If cough is present, specify how long	○ Less than 2 weeks<br>○ More than 2 weeks
if symptomatic, please specify:	○ less than 2 weeks<br>○ between 2 weeks and 1 month<br>○ between 1 - 3 months<br>○ more than 3 months

Chest US

Record ID

____

Was the Chest US performed?

◯ Yes
◯ No

Was the US performed properly?

◯ Yes
◯ No

When was performed?

____
(The CUS has to be taken within a week from diagnosis in index cases)

CUS - RIGHT LUNG

	A	B1	B2	C	P	E	U
R1	☐	☐	☐	☐	☐	☐	☐
R2	☐	☐	☐	☐	☐	☐	☐
R3	☐	☐	☐	☐	☐	☐	☐
R4	☐	☐	☐	☐	☐	☐	☐
R5	☐	☐	☐	☐	☐	☐	☐
R6	☐	☐	☐	☐	☐	☐	☐

CUS - LEFT LUNG

	A	B1	B2	C	P	E	U
L1	☐	☐	☐	☐	☐	☐	☐
L2	☐	☐	☐	☐	☐	☐	☐
L3	☐	☐	☐	☐	☐	☐	☐
L4	☐	☐	☐	☐	☐	☐	☐
L5	☐	☐	☐	☐	☐	☐	☐
L6	☐	☐	☐	☐	☐	☐	☐

FASH abdominal, positive?

◯ Yes
◯ No

Notes

____
